# Anti-Inflammatory and General Glucocorticoid Physiology in Skeletal Muscles Affected by Duchenne Muscular Dystrophy: Exploration of Steroid-Sparing Agents

**DOI:** 10.3390/ijms21134596

**Published:** 2020-06-28

**Authors:** Sandrine Herbelet, Arthur Rodenbach, Boel De Paepe, Jan L. De Bleecker

**Affiliations:** 1 Department of Head and Skin, Division of Neurology, Ghent University and Ghent University Hospital, C. Heymanslaan 10, 9000 Ghent, Belgium; Arthur.Rodenbach@UGent.be (A.R.); Boel.DePaepe@UGent.be (B.D.P.); Jan.DeBleecker@UGent.be (J.L.D.B.); 2 Neuromuscular Reference Center, Ghent University Hospital, C. Heymanslaan 10, 9000 Ghent, Belgium

**Keywords:** glucocorticoid physiology, Duchenne muscular dystrophy, skeletal muscle, steroid-sparing agents

## Abstract

In Duchenne muscular dystrophy (DMD), the activation of proinflammatory and metabolic cellular pathways in skeletal muscle cells is an inherent characteristic. Synthetic glucocorticoid intake counteracts the majority of these mechanisms. However, glucocorticoids induce burdensome secondary effects, including hypertension, arrhythmias, hyperglycemia, osteoporosis, weight gain, growth delay, skin thinning, cushingoid appearance, and tissue-specific glucocorticoid resistance. Hence, lowering the glucocorticoid dosage could be beneficial for DMD patients. A more profound insight into the major cellular pathways that are stabilized after synthetic glucocorticoid administration in DMD is needed when searching for the molecules able to achieve similar pathway stabilization. This review provides a concise overview of the major anti-inflammatory pathways, as well as the metabolic effects of glucocorticoids in the skeletal muscle affected in DMD. The known drugs able to stabilize these pathways, and which could potentially be combined with glucocorticoid therapy as steroid-sparing agents, are described. This could create new opportunities for testing in DMD animal models and/or clinical trials, possibly leading to smaller glucocorticoids dosage regimens for DMD patients.

## 1. Introduction

Duchenne muscular dystrophy (DMD) is an X-linked recessive skeletal muscle disorder affecting around one in 3500 boys. It is characterized by muscle weakness and wasting with a clinical onset at around 4 years of age. The absence of dystrophin, the protein providing a link between the extracellular matrix and the myocyte cytoskeleton, destabilizes the dystrophin-associated protein complex (DAPC), a large transmembrane protein complex that acts as a shock absorber during muscle contraction, a receiver and transducer of cellular signals and as a scaffold for signaling proteins [1,2]. Abnormal shock absorption is thought to result in increased sarcolemmal damage in the myocyte during exercise. Aberrant signal transduction in DMD is believed to result from the disturbed link between neuronal nitric oxide synthase (nNOS), dystrophin, and the DAPC. Stretch-activated channels are located downstream of dystrophin that lead to the activation of the extracellular signal regulated kinase-mitogen activated protein kinase (ERK-MAPK) cascade, along with increased Ca^2+^ influx, in DMD. Together, these pathological mechanisms induce disorganized muscle with myofiber hypertrophy and necrosis, inflammation, fibrosis, and fat deposition [3,4,5]. 

Administration of glucocorticoids (GCs) can counteract these phenomena in patients with DMD. Different GC dosing regimens can be used. The most commonly used consists of an intake of 0.75 mg/kg/day of prednisone. This is based on the strength and functional outcomes in randomized controlled trials (RCTs), as summarized in the systematic review of Matthews et al. [6]. Deflazacort is another commonly used GC. These GCs prolong ambulation and mildly improve muscle strength. The major effect of GCs is believed to reside in their inhibition of the transcription factor nuclear factor kappa-light-chain-enhancer of activated B cells (NF-κB), being the master inducer of inflammation. Unfortunately, GCs can induce several side-effects, such as suppression of the immune system, potentially leading to life-threatening infections, arrhythmias, hypertension, hyperglycemia and glucosuria, cataracts, increased food intake and weight gain, delayed growth, osteoporosis and fractures, abnormal behavior, insomnia, cushingoid appearance, easy bruising, gastro-intestinal symptoms, and hirsutism [6,7]. 

GCs regulate reproduction, development, cell differentiation, inflammation, and responses of the immune system [8]. GCs bind in the cytoplasm to the glucocorticoid receptor (GR), also known as Nuclear Receptor Subfamily 3 Group C Member 1 (NR3C1). Two isoforms termed GRα and GRβ have been described. These are the two main transcriptional isoforms [8,9,10]. While unstimulated, the GR resides mainly in the cytoplasm and is chaperoned by two heat shock protein 90 (Hsp90) molecules, Hsp90-binding protein 23 (p23), Hsp70, and immunophilins, all converging to protect the GR from degradation and prepare it for GC binding at the receptor site. Binding to the GR releases the Hsps [8,9,10]. The GR can translocate from the cytoplasm to the nucleus and back, sensing the intracellular environment along its journey and interacting with several proteins, thereby creating the GR interactome. After the binding of GCs to the GR, the actions of the transcription factor can be divided in genomic and non-genomic functions [8,9,10,11]. One of the major targets of the GR is NF-κB, the major activator of the genes coding for immunoreceptors, proinflammatory cytokines, and chemokines along with the other important cellular players inhibited by GCs. Genomic effects appear after GC–GR translocation to the nucleus and subsequent DNA binding, and can result in expression changes in up to 10–20% of genes [10]. Non-genomic actions occur within minutes and consist of the activation of Toll-like receptors (TLRs), p38 mitogen-activated protein kinases (MAPKs), c-Jun N-terminal kinases (JNKs), the NF-κB and activator protein 1 (AP-1), the Janus kinase/signal transducer and activator of transcription proteins (JAK/STAT), and transforming growth factor-β (TGF-β). 

In this review, the current evidence on the cellular pathways that are activated in and interact with GCs in the DMD myocyte, is summarized and discussed. The impact of glucocorticoid administration is visualized, thereby showing the normalization or exacerbation of the triggered pathways. Importantly, the purpose of this review is to discuss putative approaches to downscale the GC dosage in DMD patients via supplementation of the pathway stabilizing agents. All ongoing clinical trials for DMD in the USA can be monitored here: https://www.clinicaltrials.gov and in Europe on this site: https://www.clinicaltrialsregister.eu/.

## 2. Activation of the Cellular Pathways in Skeletal Muscle Cells in Duchenne Muscular Dystrophy, the Effects of GC Administration, and Putative Stabilizing Molecules

### 2.1. The TLR Signaling Pathway in DMD

#### 2.1.1. Genomic and Non-Genomic Effects of TLR Activation

**Figure 1:**
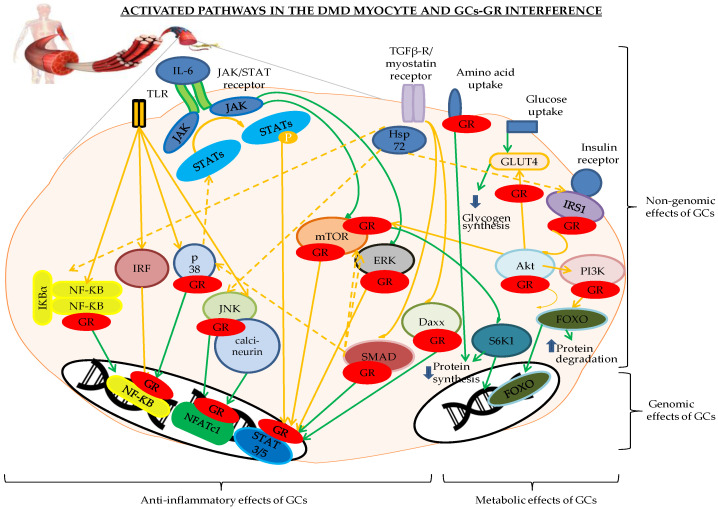
Genomic and non-genomic effects of glucocorticoid receptor (GR) binding to the different players of the inflammatory and metabolic pathways involved in the Duchenne muscular dystrophy (DMD) myocyte. The pathways indicated in orange are activated, the green indicates a return to the physiological status. The GR is depicted in red. Abbreviations: Daxx = death-associated protein, FOXO = forkhead box O, GLUT4 = glucose transporter 4, GR = glucocorticoid receptor, IκB = inhibitor of NF-κB, IRFs = interferon regulatory factors, Hsp72 = heat shock protein 72, IRS1 = insulin receptor substrate 1, JAK/STAT = Janus kinase/signal transducer and activator of transcription proteins, JNK = c-Jun N-terminal kinase, NFATc1 = nuclear translocation of nuclear factor of activated T cells c1, NF-κB = nuclear factor kappa-light-chain-enhancer of activated B cells, P = phosphorylation, p38 = p38 mitogen-activated protein kinases, PI3K/Akt/mTOR = Phosphoinositide 3-kinases/protein kinase B/mammalian target of rapamycin pathway, RAF/RAS/ERK = Rapidly Accelerated Fibrosarcoma/Ras-extracellular signal-regulated kinase, S6K1 = ribosomal protein S6 kinase beta-1, SMAD3 and -6 = Mothers Against Decapentaplegic homolog 3 and 6, TGF-β = transforming growth factor-β, TLR= Toll-like receptors.

In DMD skeletal muscle, unstable myocyte cell membranes induce TLR activation (TLR4 and TLR7), resulting in NF-κB activation through the myeloid differentiation primary response 88 (MyD88) (Figure 1) [9,10,11,12]. Furthermore, TLRs recognize the damage-associated molecular pattern (DAMP) molecules that arise from the destruction of DMD muscle fibers, such as nucleic acids, reactive oxygen species (ROS), Hsp, and very potent activators of inflammation, such as single stranded RNA (often called the ‘danger model’ of the immune system). Upon translocation of NF-κB to the nucleus, NF-κB activates the expression of proinflammatory genes coding for growth factors, cytokines, chemokines, cell adhesion molecules, and immunoreceptors [9,13]. 

A second, third, and fourth branch of the activated TLR pathway consists of interferon regulatory factors (IRFs), p38, and JNK activation. JNK is an important signaling component belonging to the group of MAPK (to which p38 also belongs). It controls proliferation and apoptosis among other functions. MAPK activation results in tumor necrosis factor (TNF) production and the production of interleukins, such as IL-1 and IL-6 [9,14,15]. Besides, JNK phosphorylates the nuclear factor of activated T cell c1 (NFATc1), a transcription factor able to activate genes coding for utrophin. NFATc1 phosphorylation results in its nuclear export [16].

Membrane instability and the ensuing TLR, NF-κB, MAPK, and IRF activation in DMD are among the major sources driving chronic inflammation, which is characteristic of the disease. As depicted in Figure 1, the major drugs able to stabilize these activated pathways are GCs by binding to NF-κB and MAPKs and through nuclear translocation of NFATc1.

#### 2.1.2. TLR Pathway Stabilizers

Lowering GC dosage regimens for DMD patients could be achieved by stabilizing parts of the activated TLR pathways. Two major modes of action could be targeted: (1) preventing the DAMPs released via myofiber destruction from binding to the TLR receptor and (2) blocking signal transduction through the activated pathways. Scanning for molecules with such properties yielded antibodies, microRNA (miRNA) inhibitors, free radical scavengers, p38 and/or JNK inhibitors, and NF-κB inhibitory peptides [15,17]. Starting with the antibodies, NI-0101 (Novimmune, Plan-les-Ouates, Switzerland) is the first anti-TLR4 antibody designed for rheumatoid arthritis (RA). With no adverse effects among healthy volunteers [16], and given that some pathways are shared between DMD and RA in pathophysiology [17,18], this antibody may be worth considering for DMD treatment. miRNAs are non-coding RNAs that can fine-tune gene expression during post-transcriptional regulation [15]. Nine miRNAs were reported to be increased in *mdx* mice, the murine model for DMD, and all are suppressed by prednisone and vamorolone (VBP15): miR-142-5p, miR-142-3p, miR-146a, miR-301a, miR-324-3p, miR455-5p, miR-455-3p, miR-497, and miR-652. Their presence in DMD skeletal muscles, their interaction with cellular pathways and if known, their specific target protein(s) are listed in Table 1. The vast majority has not been explored yet in DMD. Both miR-21 and miR-146a are specific for TLR4, and are increased in DMD skeletal muscle. miRNA-142-3p is increased in inflammatory cells and is suspected to be increased in invading inflammatory cells in DMD muscles. It interacts with glycoprotein 130 (gp130), a component of interleukin-6 receptor [15,19,20,21,22,23,24]. The muscle-enriched miRNA-206, which belongs to the so-called myomiRNAs, is increased in the serum and muscle of DMD patients [23]. It activates components involved in skeletal muscle growth and differentiation such as histone deacetylase 4 (HDAC4), polypirimidine tract-binding protein (PTB), utrophin, follistatin-like 1 (Fstl1), connexin 43 (Cx43), and the tissue inhibitor of metalloproteinases 3 (TIMP3). It inhibits insulin-like growth factor-1 (IGF-1) and paired box 3 and 7 (Pax3 and -7) [25]. The downregulation of miRNA-206 increased motor functions in *mdx* mice and provided a milder disease phenotype [26]. The inhibition of miR-21 and miR-146a could further counteract the effects of TLR4 activation in DMD. 

Another approach could involve targeting p38 MAPK and/or JNK. The treatment of *mdx* mice with alpha lipoic acid (ALA)/L-carnitine (L-Car), a free radical scavenger able to modulate p38 and JNK, resulted in decreased NF-κB activity in the *mdx* diaphragm, as listed in Table 2. It decreased the plasmatic creatine kinase level, the matrix metalloproteinase activity, NF-κB activity, antioxidant enzyme activity, and lipid peroxidation in *mdx* diaphragm [27,28]. Carnitine metabolism has been described to be perturbed in DMD. More specifically, both palmitoyl carnitine transferase and palmitoyl coenzyme A hydrolase are increased, whereas palmitoyl carnitine hydrolase is absent in DMD. The latter is an important component in carnitine metabolism and could explain the results obtained in a pilot study conducted in 2013 on a small number of steroid-naïve DMD boys with L-carnitine supplementation, showing no difference in the function of the upper and lower extremities [29,30]. An inhibitor of p38 named SB203580 provided contradictory results in *mdx* myotubes during in vitro experiments and in *mdx* mice tissue and seems to be of lesser value as a therapeutic molecule. Indeed, it prolonged survival of *mdx* myotubes in vitro under oxidative stress conditions. In *mdx* mice, the p38 MAPK phosphorylation levels were normal [27,31]. Another study on *mdx* mice with the JNK1 inhibitory protein (JIP1) showed attenuation of muscle fiber necrosis [32]. Deflazacort, an oxazoline derivative of prednisone, enhances the transcription of the utrophin gene, thereby compensating in part for the loss of dystrophin by upregulating the activity of calcineurin phosphatase through JNK1. This leads to the nuclear translocation of NFATc1, a stimulator of the utrophin gene [16]. JIP1 seems promising because it increases *mdx* myotube viability in vitro and decreases *mdx* myofiber destruction in vivo. However, further studies are needed [33]. The direct inhibition of IRF in DMD has not been described to date; all reported IRF inhibitions were indirect [34,35].

An important effector of the TLR pathway is the proinflammatory transcription factor NF-κB (60 kDa), which is activated in DMD [36]. Many molecules have been tested to target this master regulator of inflammation (Table 3). In *mdx* myotubes or mice, several studies were performed with NF-κB inhibitors, such as NK-κB Essential MOdulator (NEMO)-Binding Domain (NBD), the antioxidant pyrrolidine dithiocarbamate (PDTC), the inhibitor of lipid peroxidation IRFI-042, and the free radical scavengers, N-acetylcysteine (NAC) and (ALA)/L-carnitine (L-Car) [31,37,38,39,40,41,42]. Unfortunately, NBD induced renal toxicity in *mdx* mice despite encouraging results showing decreased necrosis and increased regeneration in the diaphragm and hind limb muscles [37,43,44]. Administration of the NF-κB inhibitor PDTC showed increased strength and muscle regeneration along with decreased fatigue and muscle necrosis in *mdx* mice. In the diaphragms of *mdx* mice, PDTC influenced the diameter and density of myofibers [45,46]. Several other NF-κB inhibitors can also be considered. IRFI-042 showed a decrease in necrosis and an increase in regeneration in *mdx* muscle [46]. While NAC decreased the membrane permeability and increased the stretch-induced force in *mdx* muscle, as well as decreased myofiber necrosis in an *mdx* diaphragm, it caused severe side-effects, such as a marked drop in body and liver weight [47,48,49,50,51,52]. Edasanolexent (CAT-1004, Catabasis, Cambridge, MA, USA) an NF-κB inhibitor, improved resistance to eccentric contraction-induced damage in *mdx* muscle [53]. The Phase I/II trial (Move DMD^®^, NCT02439216) reported minor side-effects, such as mild gastrointestinal symptoms and headache. Inhibition of NF-κB was noticed. The results from the Phase II clinical trial are pending, whilst Phase III (PolarisDMD, NCT03703882) and Phase III open label studies (GalaxyDMD, NCT03917719) are currently ongoing [54,55,56]. AP-1 is always mentioned in the same breath with NF-κB in GC administration. It is an activator of utrophin [57]. Batimastat (BB-94) inhibited AP-1 in *mdx* muscle [58]. AP-1 has been poorly studied in DMD. One study reported its upregulation in *mdx* muscle tissue [59], one pinpointed its importance in skeletal muscle regeneration [60], and one linked it to oxidative stress in skeletal muscle cells [61]. Inhibition of AP-1 by batimastat would imply absence of utrophin activation and seems less interesting as a therapeutic option in DMD. 

### 2.2. The JAK/STAT Signaling Pathway in DMD

#### 2.2.1. Genomic and Non-Genomic Effects of JAK/STAT Activation

The JAK/STAT pathway is involved in skeletal muscle physiology and pathology. Skeletal muscles are producers of IL-6, thereby earning the title of myokine, which has warning potential towards other organs, such as the pancreas and liver, resulting in glucose tolerance and production while harboring anti-inflammatory properties. The binding of cytokine IL-6 to its receptor (IL-6R) and to the protein gp130 homodimer results in the activation of JAK and STAT3, which subsequently translocates to the nucleus. In DMD, IL-6 production is increased and plays a proinflammatory role, leading to the exhaustion of satellite cells [62,63]. In *mdx* mice, STAT3 and IL-6 are upregulated. Moreover, the chronic IL-6 elevation produced by T cells and macrophages, as part of the chronic inflammation seen in DMD, plays an important role in the muscle pathology leading to the activated JAK/STAT pathway and NF-κB [64]. However, the expression patterns of STAT3 and IL-6 are different in individual *mdx* muscles, leading to aggravation of the phenotype in one muscle, while amelioration is observed in another muscle [65,66]. GCs and IL-6 synergistically activate the IL-6 response element through GR and STAT3 [63,67]. The JAK/STAT pathway directly communicates through STAT3 and the suppressor of cytokine signaling 3 (SOCS3) with the Rapidly Accelerated Fibrosarcoma/Ras-extracellular signal-regulated kinase (RAF/RAS/ERK) and the Phosphoinositide 3-kinases/protein kinase B/mammalian target of rapamycin (PI3K/Akt/mTOR) pathways [64,65] (Figure 1). These pathways are cell controlling towers for cell survival, motility, and proliferation after extracellular signaling. In *mdx* mice, these pathways are activated to increase the expression of α7 integrin and compensate for the loss of dystrophin [66].

#### 2.2.2. JAK/STAT Pathway Stabilizers

As mentioned above, the versatile function of JAK/STAT in cell signaling makes it a difficult target in pathway stabilization. The controversial results observed in *mdx* mice after using an antibody directed against IL-6 (tocilizumab, RoActemra^®^, Roche, Basel, Switzerland) requires careful consideration before it can be part of the list of putative molecules for DMD treatment (Table 4). For *mdx* mice, one study reported a decrease in *mdx* muscle inflammation, while another study described an increase in inflammation [64,66,68,69]. Valproic acid (VPA) has been described to inhibit apoptosis by activating the PI3K/Akt/mTOR pathways in *mdx* mice. It ameliorated the *mdx* phenotype by increasing the sarcolemmal integrity, and decreasing fibrosis and hind limb contractures in *mdx* muscle and mice [70]. It may be worth considering VPA supplementation for DMD patients on a GC regimen.

### 2.3. The TGF-β Signaling Pathway in DMD

#### 2.3.1. Genomic and Non-Genomic Effects of TGF-β Activation

After activation, TGF-β phosphorylates the Mothers Against Decapentaplegic homolog 3 and 6 (SMAD3 and -6) and interacts with the death-associated protein Daxx. Two phosphorylated SMAD3s bind to an SMAD4. This complex subsequently translocates to the nucleus. After translocation to the nucleus, the GR will prevent binding SMAD3, 4, 6, and Daxx to the DNA [9]. SMAD plays an important role in muscle cell growth and differentiation [71]. The T- and B cells present in DMD muscle, along with the DMD myotubes themselves, secrete TGF-β. At high doses, TGF-β can induce fibrosis in skeletal muscle by stimulating fibroblasts and enhancing a switch to the myofibroblast phenotype, thereby prompting the deposition of the extracellular matrix (ECM), pitchforking it to the ‘master regulator of fibrosis’. In contrast, low doses of TGF-β stimulate the proliferation of myoblasts to develop them into fast-type muscle fibers. Indeed, the suppression of TGF-β1 during skeletal muscle regeneration induces the appearance of slow-type MHC, thus guiding regeneration towards the slow muscle fiber type phenotype. Myostatin is intrinsically linked to TGF-β [72]. TGF-β/myostatin activation has been widely studied in DMD [71,72,73,74,75,76]. GCs bind to SMAD and Daxx, and can interfere with satellite cell differentiation, which is detrimental to DMD patients. Chronic GC use has been shown to reduce fibrosis in DMD muscle [77].

#### 2.3.2. TGF-β/Myostatin Pathway Stabilizers

TGF-β and myostatin inhibitors comprise the majority of molecules currently being/have been tested in DMD clinical trials (Table 5) [78]. Two TGF-β inhibitors are losartan and suramin. Losartan (Cozaar^®^, MSD, Kenilworth, NJ, USA) is an angiotensin II type 1 receptor blocker used in the treatment of high blood pressure. It reduces the expression of TGF-β and the production of connective tissue growth factor (CTGF) [79]. When used in *mdx* mice, losartan inhibited fibrosis in the *mdx* muscle and decreased fibrosis and increased muscle fiber density in the *mdx* diaphragm, while another study on *mdx* mice did not show any difference in skeletal muscle [78,80,81]. Suramin (Germanin^®^, Bayer, Leverkusen, Germany), is an anti-parasitic and anti-neoplastic FDA approved molecule, used to treat African sleeping sickness and river blindness or treat some sorts of cancer. It interacts with TGF-β by preventing binding to the TGF-β receptor [82]. Suramin was shown to reduce fibrosis and myofiber necrosis in *mdx* mice [83]. The proteoglycan decorin has the ability to trap both TGF-β and myostatin, with interesting results showing fibrosis downscaling (collagen type I levels in the diaphragm) and the enhancement of muscle regeneration in *mdx* mice [71,73,74]. Another strategy consists of targeting activin, one of the members of the TGF-β superfamily, which binds to the activin receptors IIA and IIB (ACVR2A, ACVR2B). The soluble activin receptor ACE-031 (Acceleron Pharma, Cambridge, MA, USA) ActRIIB-IgG was tested, due to increased forward pulling tension in *mdx* mice [84], in a Phase II trial on DMD patients (2011). The patients showed tolerance towards the molecule with only minor side effects. However, these side effects resulted in the termination of the study by the USA FDA, despite an increase in muscle mass [85]. *Acvr2b* downregulation by the AAV-virus delivery of shRNAs induced *mdx* muscle hyperplasia [86]. The bone morphogenic proteins (BMP) are a group of growth factors which enhance muscle growth and play a role in muscle disorders [87]. Therefore, the following BMP antagonists could be considered: dorsomorphin, LDN-193189, and Noggin. However, dorsomorphin is toxic to in vitro grown primary myoblasts [88]. Noggin alleviated the dystrophic phenotype in *mdx* mice [88]. Tamoxifen (Hadassah Medical Organization, Ein Kerem, West Jerusalem), a selective estrogenreceptor modulator, stabilized myofiber membranes, normalized the whole body force, and reduced fibrosis in the diaphragm in *mdx* mice [89,90,91]. A Phase I clinical trial (NCT02835079) is currently ongoing in Israel, with completion expected in November 2020. A Phase clinical trial III (TAMDMD, NCT03354039, EudraCT Number: 2017-004554-42) is ongoing in Europe and should be completed in June 2020. Patients receive in both trials also GCs [92]. The antiparasitic molecule halofuginone (HT-100) inhibited *mdx* muscle and diaphragm fibrosis [93,94]. All Phase I/II/III clinical trials in the USA (HALO-DMD-01, NCT01847573; HALO-DMD-02, NCT01978366; HALO-DMD-03, NCT02525302) were abrogated after the death of a patient on a high dose of HT-100. Halofuginone and GCs tend to work in a synergistic manner. Safer molecules derived from halofuginone such as deoxyhalofuginone have been tested in *mdx* mice, with positive effects on muscle fiber diameter [95]. 

Myostatin targeting antibodies include domagrozumab (PF-06252616, Pfizer, New York, NY, USA), talditercept alpha (anti-myostatin adnectin, RG 6206, BMS-986089, Bristol-Myers Squibb, New York, NY, USA), and stamulumab (MYO-029, Wyeth Pharmaceuticals, Madison, NJ, USA). A clinical trial with stamulumab over six months in Becker muscular dystrophy (BMD) patients (completed in January 2007) showed good safety but no increase in skeletal muscle strength [96]. Domagrozumab increased functional muscle mass and increased body and muscle weight and grip strength. The size of the tibialis anterior fibers was significantly increased in *mdx* mice [97]. All clinical trials (NCT02310763 and NCT02907619) were abrogated in 2018 due to a lack of effectiveness [71,76,78,98]. Currently, talditercept alpha is being tested in an ongoing Phase I/II trial in the USA (NCT02515669, THUNDERJET), which should be completed in May 2020, and a worldwide Phase II/III trial (NCT03039686, SPITFIRE) due in December 2024. It is worth mentioning that myostatin seems to be associated with the GCs induced catabolic effect on muscle proteins and less with the suppression of protein synthesis [99]. The myostatin inhibitory protein follistatin (rAAV1.CMV. huFollistatin344, Nationwide Children’s Hospital, Columbus, OH, USA) showed an increase in muscle mass, myofiber size, grip strength, and tetanic force, as well as a decrease in necrosis, fibrosis, and inflammation, in *mdx* mice [100,101,102,103]. Follistatin was tested in a Phase I/II clinical trial (NCT02354781) completed in 2017, with good results. The TGF-β pathway can also be modulated by the knockdown of the TGF-β Type I receptor TGFBR1, also known as activin A receptor type II-like kinase 5 (ALK5), via the exon skipping of ALK5 by specific antisense oligonucleotides (AONs) [104]. TGF-β activation in DMD induces the upregulation of CTGF [105]. With GCs inducing CTGF, a molecule was developed that can downregulate CTGF: Pamrevlumab (FG-3019, Fibrogen, San Francisco, CA, USA). This molecule increased muscle strength and endurance and induced a decrease in apoptosis and fibrosis in *mdx* muscle [106]. Currently, a Phase II clinical trial (NCT02606136) is ongoing in the USA, with completion expected in April 2021. 

### 2.4. The Metabolic Pathways in DMD

#### 2.4.1. Genomic and Non-Genomic Effects of Metabolic Pathway Activation

Insulin regulates glucose uptake in the skeletal muscle. DMD muscle presents disturbed insulin signaling resulting in insulin resistance (IR), possibly due to an abnormal cellular localization of glucose transporter 4 (GLUT4) (Figure 1). Decrease in molecular chaperone protein Hsp72 plays a role in IR in skeletal muscle. Besides, it also inhibits JNK and NF-κB. In DMD, the exact status of Hsp72 is not clear cut and IR occurs independently of GC intake [106,107,108,109]. However, when taken for a longer period of time, GCs will further induce IR. GCs can also induce myopathy through oxidative stress and the production of factors to counteract this stress. When examining the glucocorticoid sensitivity in muscle, fast-twitch myofibers have a larger GR density than slow-twitch myofibers, making them more susceptible to GC toxicity [110,111]. The brain is the largest consumer of glucose and needs to be fueled in a permanent manner, especially in times of stress. Therefore, the major role of GCs in skeletal muscle metabolism is blood glucose homeostasis by reducing both glucose uptake and consumption by the muscles. Further, GCs support catecholamine-induced glycogenolysis and inhibit the insulin-stimulated synthesis of glycogen in the muscle. GCs also promote the degradation of protein in the muscle, thereby facilitating gluconeogenesis by providing gluconeogenic amino acids in the muscle during the synthesis of its own glucose. This keeps glucose in the blood to fuel the brain during stress. Insulin, on the contrary, exerts opposing effects that enhance glucose utilization by the muscle [109,111]. In myotubes, GCs reduce insulin-stimulated glucose uptake by diminishing the translocation of GLUT4 to the cell membrane. This also results in decreased glycogen synthesis. GCs also block the insulin-receptor-Akt-mTOR-S6K pathway and the insulin-receptor-Akt-FOXO pathway, culminating in an increase of protein degradation and a decrease of protein synthesis. After insulin binding to the insulin receptor, GCs block the downstream pathway at the levels of insulin receptor substrate 1 (IRS1), phosphoinositide 3-kinase (PI3K), protein kinase B (PKB/Akt), mammalian target of rapamycin (mTOR), ribosomal protein S6 kinase beta-1 (S6K1), and forkhead box O (FOXO). GCs block amino acid uptake in muscles. All these modulations by GCs converge into hyperglycemia in the blood, fueling the brain with glucose during stressful events (Figure 1) [109,110,111,112]. 

#### 2.4.2. Glucose Metabolism Stabilizers

Glucose intolerance and significant changes in blood glucose have been described in DMD patients [113,114], and glucose metabolism has also been described as a disease marker in the Golden Retriever Model of DMD [115]. With chronic GC intake inducing IR, it may be worth considering the use of insulin sensitizers in DMD patients (Table 6). Insulin sensitizers address IR by increasing the sensitivity of the organs to insulin. The biguanide metformin (Glucophage^®^, Fortamet^®^, Riomet^®^) induces glucose uptake in skeletal muscle, is widely used in children and teenagers with diabetes type 2, is safe to use in patients with heart failure, and does not induce weight gain [116]. Metformin was tested in *mdx* mice and showed reduced fibrosis in the *mdx* muscle but no effect on glucose metabolism [117]. It increased twitch and tetanic tension in *mdx* diaphragm. In vitro, metformin protected the myotubes from cardiotoxin induced degeneration [118,119]. A Phase I clinical trial (NCT02516085) completed in 2012 in Switzerland showed slowing of the disease process in DMD patients but no change in motor function [120,121]. Adiponectin is decreased in *mdx* mice and DMD patients [122,123] and is widely recognized as an insulin sensitizer. Studies on AdipoRon (adiponectin) in *mdx* mice revealed a delay in the disease progression, reduced inflammation, an increase in anti-inflammatory cytokine IL-10, protection from T-lymphocyte and M1 macrophage infiltration, a transition to the M2 macrophage type, and a boost of the myogenic program. Similar observations were made in DMD human myotubes treated with AdipoRon [123,124,125,126]. The pharmacological co-inducer of Hsp72, BGP-15, showed promising results in *mdx* mice by increasing muscle strength, architecture, and contractile function in the diaphragm when given at an early age [109,127]. Idebenone was created for the treatment of Alzheimer’s disease and could be considered an insulin sensitizer [128]. In *mdx* mice, idebenone improved running performance [129]. Two Phase II clinical trials (NCT00654784 and NCT00758225, Delphi extension) were completed in 2007 and 2011 in Belgium; a worldwide Phase III clinical trial (NCT01027884) was also finished in 2014. These trials showed improved respiratory function in DMD patients [130,131,132]. Two phase III clinical trials are ongoing: NCT02814019, SIDEROS expected in August 2021, and NCT03603288, SIDEROS-E expected in January 2024. 

### 2.5. The Histone Deacetylases HDAC in DMD

#### 2.5.1. Genomic and Non-Genomic Effects of HDAC Activation in DMD

**Figure 2:**
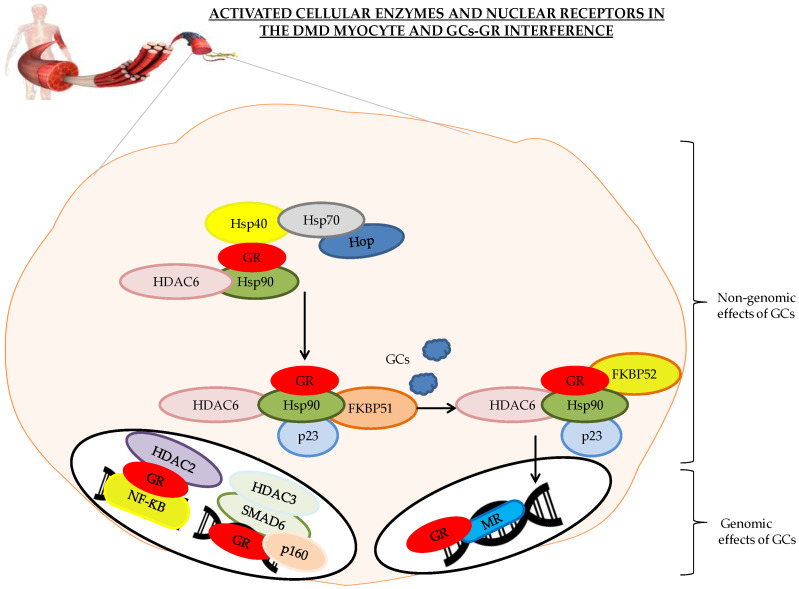
Genomic and non-genomic effects of the glucocorticoid receptor (GR) binding to activated cellular enzymes and the nuclear receptors involved in the Duchenne muscular dystrophy (DMD) myocyte. The GR is depicted in red. Abbreviations: FKBP51 and -52 = immunophilin FK506-binding protein 51 and 52, GCs = glucocorticoids, HDAC3 and -6 = Histone deacetylase 3 and -6, hsp90 = heat shock protein 90, Hop = Hsp70–Hsp90 organizing protein, MR = Mineralocorticoid receptor, SMAD6 = Mothers Against Decapentaplegic homolog 6.

As depicted in Figure 2, several steps of preparation are necessary for GCs to bind to GRs in the cytoplasm. HDAC6 needs to bind to Hsp90. The deacetylation of Hsp90 provides stable binding to the GR, followed by the release of the Hsp70-Hsp90 organizing protein (Hop), Hsp40, and Hsp70, and the stabilization of Hsp90 by cochaperone p23 and immunophilin FK506-binding protein 51 (FKBP51), a potent inhibitor of GR [133]. Therefore, p23 and FKBP51 are both necessary to form a stable chaperone- and cochaperone heterocomplex with GR. After GCs binding to the GR heterocomplex, it is believed that, FKBP51 is replaced by FKBP52, subsequently potentiating GR [134,135]. After translocation to the nucleus, GR binds to the glucocorticoid response elements (GREs) in the DNA and activates anti-inflammatory genes [9]. The absence of HDAC6 blocks the transfer of the GR to the nucleus via the creation of an unstable Hsp/GR complex. Further, HDAC2 helps deacetylate GRs in the nucleus, resulting in a decrease in the transcription of inflammatory genes after NF-κB binds to the DNA. During the interaction of SMAD6 with the GR, HDAC3 is recruited, leading to decreased transcription of GR genes [9]. In *mdx* mice, HDACs are upregulated [136].

#### 2.5.2. HDAC Inhibitors

HDAC inhibitors (HDACis) upregulate follistatin in dystrophic muscle, thereby improving regeneration. Indeed, HDACis achieve this by inhibiting the activity of Myogenin D (MyoD) [137]. The orphan drug Givinostat (S2170, formerly known as ITF2357) (Table 7) is an HDAC inhibitor that increases the cross-sectional myofiber area, membrane stability, and endurance, and decreases fibrosis and inflammation in *mdx* muscle [138,139,140]. A Phase I/II trial (NCT01761292) in DMD patients between 8 and 10 years of age (as HDACi only has an impact early in the disease) was completed in 2017 in Italy, showing good tolerance and a decrease in necrosis, fibrosis, and fatty tissue [141]. Notably, biopsies are needed to evaluate the progression of the disease under an HDACi regimen, which involves subjecting patients to an invasive procedure [142]. In Europe and the USA, Phase II/III (NCT03373968) and III (NCT02851797) trials are ongoing, with completion expected in December 2023 and March 2022, respectively. HDACis can cause side-effects such as arrhythmias, nausea, vomiting, anorexia, fatigue, anemia, and liver toxicity [143]. When HDACis are combined with VPA, status epilepticus has been described [143]. Therefore, HDACis should be handled very cautiously in DMD.

### 2.6. The Activation of Nuclear Receptors in DMD 

Mitochondria are involved in the pathogenesis of DMD, by exhibiting lower expression of mitochondrial genes along with disturbed oxidative phosphorylation. Peroxisome proliferator-activated receptor γ coactivator 1-alpha (PGC1α) plays an important role in regulating the oxidative metabolism. When overexpressing PGC1α in *mdx* mice, increased mitochondrial biomass is noticed along with improved Ca^2+^ handling in mitochondria in *mdx* muscle. Therefore, the nuclear receptors peroxisome proliferator-activated receptor α and γ (PPARα and –γ) could be exploited as therapeutic targets in DMD (Table 8) [144]. During inflammation, GCs collaborate with PPARα and -γ to diminish chemokines [9]. The activation of PPARβ/δ in *mdx* mice by the PPARβ/δ agonist GW501516 stimulates utrophin A expression and restores sarcolemmal integrity [144,145]. Another nuclear receptor, mineralocorticoid receptor (MR), involved in skeletal muscle tissue protection, can promote NF-κB during inflammation, which is counteracted by GCs binding to MRs. The MR-binding vamorolone was shown to improve muscle function in DMD with fewer side-effects [11,146]. It increased membrane stability, grip strength, and force, and also decreased inflammation, in *mdx* muscle [11]. Two Phase II trials (NCT02760264 and NCT02760277) were conducted worldwide and completed in 2018, showing good tolerance and improved muscle function in DMD boys [147]. Two Phase II trials (NCT03038399 and NCT03439670) are ongoing worldwide and should have been completed, respectively, in April and May 2020. 

## 3. Discussion

In Table 1, Table 2, Table 3, Table 4, Table 5, Table 6, Table 7 and Table 8, summaries are provided, along with the outcomes, of finalized clinical trials using stabilizing agents in DMD patients, as well as ongoing trials with their expected due dates [147]. Besides, results obtained with these agents in *mdx* mice are included. For all studies conducted on *mdx* mice, one should keep in mind that these mice express a milder phenotype than that of DMD patients, and extrapolation to the human condition should be done with care. The reasons for this phenomenon reside in the ability of *mdx* mice to produce utrophin along with the presence of a high regenerative capacity in their satellite cells [148].

When considering a polypharmaceutical approach for DMD patients, it is of the utmost importance to know which molecules should never be combined with GCs. Cytochrome P450 3A4 (CYP3A4) inhibitors, such as cobicistat (Gilead Sciences, Foster City, CA, USA), should not be administered alongside GCs [149]. The combination of GCs with glucose lowering drugs, such as metformin, requires great caution. It should be kept in mind that myostatin seems to be associated with the GCs induced catabolic effect on muscle proteins [97] and HDACis can cause severe side-effects, especially when combined with VPA [143]. In the past, a high dosage regimen of halofuginone, an anti-parasitic molecule used in veterinary medicine, combined with GCs led to the death of a DMD patient and abrogation of the clinical trial in 2016 [77]. One possible explanation could be found in the synergy between halofuginone and GCs [90,91,92,93]. When considering polypharmacy in DMD, exploring molecular interactions is of great importance in avoiding life-threatening drug combinations.

From the therapeutic armamentarium tested in *mdx* mice and in DMD patients, the vast majority are molecules which were primarily designed for other diseases such as RA, Alzheimer’s disease, and regulation of the blood pressure or glucose homeostasis, to name a few. When blocking an entire pathway, such as the TGF-β, TLR, JAK/STAT or the glucose metabolism pathway, many other functions in the cell will be affected with repercussions in the cellular activity, which can be noticed as side-effects in patients (Table 1, Table 2, Table 3, Table 4, Table 5, Table 6, Table 7 and Table 8). Inhibition of the TGF-β pathway seems to often produce side-effects (Table 5) [85,92,93,94,95]. Although some molecules have not yet been tested in clinical trials with DMD patients, such as losartan, suramin, and decorin, the potential side-effects make these molecules less attractive. The TGF-β cellular pathway is involved in many cellular functions ranging from cell growth to apoptosis and should be considered as a cellular ‘highway’ which upon blocking is likely to yield side-effects. Therefore, anti-TGF-β signaling drugs are challenging in finding a therapeutic window and dosing regimen still efficacious and with only minor side effects [150]. Follistatin is an autocrine glycoprotein expressed on nearly all cells of the body and side-effects were noticed during the Phase I/II trial (NCT02354781). Inhibition of myostatin, a myokine, is muscle specific. Usage of antibodies against myostatin will reveal in the near future their therapeutic value. The first outcome of the Phase II (NCT02606136) clinical trial currently ongoing in the USA, should be known after completion of the trial in April 2021.

Although the TLR-pathway is more oriented on the inflammatory response in skeletal muscle [151], many downstream proteins have broad cellular function, especially MAPK, which plays an important role in cellular proliferation [152]. From the TLR inhibitors listed as putative stabilizing agents, NI-0101 was used in RA in a Phase II clinical trial and did not show any improvement in the disease parameters [153]. Therefore, it may be less interesting to focus on this antibody. When considering miRNAs as a therapeutic option in DMD, cautiousness is required as miRNAs can harbor several modes of action. miR-146a deficiency in *mdx* mice did not aggravate the phenotype, but a slight increase in muscle damage and inflammation was observed [154]. Therefore, inhibiting miR-146a should be considered very carefully. Caution is also required when considering miR-21 inhibition. Indeed, in *mdx* mice, miR-21 is increased in fibroblasts and downregulated in myoblasts [19], presumably making it a difficult target. miR-206 is known for activating a large array of proteins such as HDAC4, PTB, utrophin, Fstl1, Cx43, and TIMP3 and for inhibiting IGF-1, Pax3, and Pax7 [25]. Despite promising results in *mdx* mice with (ALA)/L-carnitine (L-car) and its FDA approval, the absence of palmitoyl carnitine hydrolase in DMD muscle and its essential role in carnitine metabolism, makes this molecule uninteresting to consider as a stabilizing agent as it did not show any difference in function of extremities in DMD patients [29,30,31]. Considering the JNK inhibitor JIP1 is less interesting as it is generally administered through adenoviral infection, which makes it less suitable. The NF-κB inhibitors PDTC and IRFI-042 and the AP-1 inhibitor batimastat (Table 3) can all be orally ingested. However, batimastat displays a low oral bioavailability [155]. Neither PDTC nor IRFI-042 are FDA approved and could therefore not be considered for clinical trials in conjunction with GCs [45,46,47,48,49,50,51,52,56]. The JAK/STAT pathway stabilizing agent VPA, on the other hand, is FDA approved (ANDA 073229). It activates the PI3K/Akt/mTOR pathway and reduces apoptosis. It may be worth considering for DMD clinical trials yet keeping in mind it should never be combined with HDACis. This combination can trigger status epilepticus [70,143]. Potential danger for DMD patients could also arise when combining glucose lowering molecules with GCs, which should be avoided at any time (Table 6). Less dangerous, but still detrimental, are the side-effects seen with HDACis (Table 7). With arrhythmias, nausea, vomiting, anorexia, fatigue, anemia, and liver toxicities having been described, HDACis should be considered very cautiously in DMD [143]. Therefore, it is understandable that the scientific community has high hopes for vamorolone. Nevertheless, the cross-talk between the inflammatory and metabolic pathways through p38 MAPK, mTOR, and Akt, as depicted in Figure 1, could lead to the combination of several stabilizing molecules in conjunction with GCs and a subsequent decrease in GC-dosing. The theoretical combination of GCs with VPA should first be tested in *mdx* mice. If beneficial effects are observed, a polypharmaceutical approach could be applied in a clinical trial, potentially leading to smaller GC regimens for DMD patients, which would be beneficial for their health. 

Many research efforts are put in gene and cell therapies for DMD. To name some gene therapies: exon skipping, stop codon readthrough, AAV-mediated therapies, RNA interference, clustered regularly interspaced short palindromic repeats (CRISPR/Cas9), all with the intention to restore dystrophin production in the DMD myocyte [156,157,158,159,160]. Cell therapies consist of autologous or allogenic muscle stem cell implantation in DMD patients [161]. A mixed approach of gene therapy with cell therapy along with GCs intake and supplementation of a steroid sparing agent such as VPA, could be worth considering. The cellular perturbations due to the lack of dystrophin and instable DAPC need to be addressed along with restoring dystrophin expression by gene therapy. Polypharmacy in DMD should always be applied after careful study of potentially life-threatening side-effects when combining several molecules, to safeguard the life of our DMD patients.

## Figures and Tables

**Table 1:** Overview of miRNAs in Duchenne muscular dystrophy (DMD), their influence on other cellular pathways and their target protein.

Molecule	Presence in DMD	Involved Pathway + Target Protein(s)
miR-21	Increased in skeletal muscle	TLR4 [16]
miR-142-5p	Unknown	-
miR-142-3p	Increased in inflammatory cells, probably also during skeletal muscle invasion [22]	gp130 [24]
miR-146a	Increased in skeletal muscle [23]	TLR4 [16]
miR-206	Increased in skeletal muscle [23]	Activation of HDAC4, PTB, Utrophin, Fstl1, Cx43, TIMP3. Inhibition of IGF-1, Pax3, and Pax7 [25].
miR-301a	Unknown	-
miR-324-3p	Unknown	-
miR455-5p	Unknown	-
miR-455-3p	Unknown	-
miR-497	Unknown	-
miR-652	Unknown	-

**Table 2:** Overview of p38 mitogen-activated protein kinases (p38 MAPK) and c-Jun N-terminal kinase (JNK) stabilizing molecules: results in *mdx* myotubes (in vitro) or mice (in vivo), finished clinical trials and results, ongoing clinical trials and due dates, and putative molecules.

Molecule	Involved Pathway	Results in *mdx* Myotubes or Mice	Finished Clinical Trials + Results	Ongoing Clinical Trials + Due Date
Free radical scavenger lipoic acid (ALA)/L-carnitine (L-Car)	p38 MAPK modulation	Decreased plasmatic creatine kinase level, matrix metallo- proteinase activity, NF-κB activity, antioxidant enzyme activity and lipid peroxidation in *mdx* diaphragm [27,28]	-	-
p38 inhibitor SB203580	p38 MAPK modulation	prolongs survival of *mdx* myotubes in vitro under oxidative stress conditions but in *mdx* mice [27,31]	-	-
JNK1 inhibiting protein (JIP1)	JNK inhibition	Increased *mdx* myotube viability in vitro and decreased *mdx* myofiber destruction in vivo [33]	-	-

**Table 3:** Overview of nuclear factor kappa-light-chain-enhancer of activated B cells (NF-κB) and activator protein 1 (AP-1) stabilizing molecules: results in *mdx* myotubes (in vitro) or mice (in vivo), finished clinical trials and results, ongoing clinical trials and due dates, and putative molecules.

Molecule	Involved Pathway	Results in *mdx* Myotubes or Mice	Finished Clinical Trials + Results	Ongoing Clinical Trials + Due Dates
NK-κB Essential MOdulator (NEMO)-Binding Domain (NBD)	NF-κB inhibition	Decreased necrosis and increased regeneration in diaphragm + hind limb *mdx* mice. Induces **kidney toxicity** in *mdx* mice [37,43,44]	-	-
Pyrrolidine dithiocarbamate (PDTC)	NF-κB inhibition	Increased strength and muscle regeneration and decreased fatigue and muscle necrosis in *mdx* mice [45]. Influence on myofiber diameter, density, and survival in *mdx* diaphragm tissue [46,47]. Whole body tension is increased in *mdx* mice [48].	-	-
Inhibitor of lipid peroxidation IRFI-042	NF-κB inhibition	Decrease in necrosis and increase in regeneration in *mdx* muscle [48,49,50,51,52]	-	-
free radical scavengers N-acetylcysteine (NAC)	NF-κB inhibition	Increase in stretch-induced force in *mdx* muscle, decrease in myofiber necrosis in the *mdx* diaphragm, severe drop in body and liver weight [51,52]	-	-
Edasalonexent (CAT-1004)	NF-κB inhibition	Improved resistance to eccentric contraction-induced damage in *mdx* muscle [53,54]	Phase I/II (Move DMD^®^, NCT02439216) in USA, with very mild gastrointestinal side-effects and headache and NF-κB inhibition. Phase II results pending. **GC intake was an exclusion criterium** [55].	Phase III (PolarisDMD, NCT03703882) and Phase III open label (GalaxyDMD, NCT03917719), USA. Completion expected in June 2020 and June 2022. **GC intake was an exclusion criterium**.
Batimastat (BB-94)	AP-1 inhibition	Reduction of proinflammatory AP-1 expression in *mdx* muscle [56]	-	-

**Table 4:** Overview of Janus kinase/signal transducer and activator of transcription proteins (JAK/STAT) and mammalian target of rapamycin pathway (mTOR) stabilizing molecules: results in *mdx* myotubes (in vitro) or mice (in vivo), finished clinical trials and results, ongoing clinical trials and due dates, and putative molecules.

Molecule	Involved Pathway	Results in *mdx* Myotubes or Mice	Finished Clinical Trials + Results	Ongoing Clinical Trials + Due Dates
Tocilizumab (RoActemra^®^)	IL-6R antagonist	Decrease and increase in *mdx* muscle inflammation [64,66]	-	-
valproic acid (VPA)	PI3K/Akt/mTOR pathways activator	Increase in sarcolemmal integrity and decrease in fibrosis in *mdx* muscle [70]	-	-

**Table 5:** Overview of tumor growth factor β (TGF-β) or myostatin stabilizing molecules: results in *mdx* myotubes (in vitro) or mice (in vivo), finished clinical trials and results, ongoing clinical trials and due dates, and putative molecules.

Molecule	Involved Pathway	Results in *mdx* Myotubes or Mice	Finished Clinical Trials + Results	Ongoing Clinical Trials + Due Dates
Losartan (Cozaar^®^)	TGF-β inhibition	Reduction in fibrosis in *mdx* muscle, increased muscle fiber density, and decreased fibrosis in the *mdx* diaphragm [78,80,81]	-	-
Suramin (Germanin^®^)	TGF-β inhibition	Reduction in fibrosis and myofiber necrosis in *mdx* mice [83]	-	-
Decorin	TGF-β inhibition	Fibrosis downscaling in the diaphragm and enhancement of muscle regeneration in *mdx* mice [71,73,74]	-	-
ACE-031	TGF-β inhibition	Increased forward pulling tension in *mdx* mice [84]	Phase II trials (NCT01099761 and NCT01239758) were completed in 2011 with good tolerance and increase in muscle mass. Abrogated due to side effects [85].	-
*Acvr2b* downregulation by AAV-virus delivery of shRNAs	TGF-β inhibition	Induction of *mdx* muscle hyperplasia [86]	-	-
BMP antagonists: dorsomorphin, LDN-193189 and Noggin	TGF-β inhibition	Dorsomorphin is **toxic** in in vitro grown primary myoblasts. Noggin alleviates the dystrophic phenotype in *mdx* mice [88].	-	-
Tamoxifen	TGF-β inhibition	Stabilization of myofiber membranes, normalization of whole body force and reduction of fibrosis in the diaphragm in *mdx* mice [89,90,91]	-	Phase I (NCT02835079) conducted in Israel. Completion expected in November 2020. Phase III trial (TAMDMD, NCT03354039, EudraCT Number: 2017-004554-42) ongoing in Europe, with completion expected for June 2020. **All patients also receive GCs** [92].
Halofuginone (HT-100)	TGF-β inhibition	Inhibition of *mdx* muscle and diaphragm fibrosis [93,94]	Phase I/II trial (HALO-DMD-01, NCT01847573) in USA, abrogated in 2016. Phase II trial (HALO-DMD-02, NCT01978366) in the USA, abrogated in 2016. Phase III trial (HALO-DMD-03, NCT02525302) in the USA, abrogated in 2016. **Patients also received GCs**.	-
Domagrozumab (PF-06252616)	Myostatin inhibition	Increase in functional muscle mass and normal neuromuscular coordination in *mdx* mice [97]	Phase II trial (NCT02310763) + open-label extension study (NCT02907619) in the USA were abrogated in 2018 (no effectiveness). **Patients also received GCs**.	-
Talditercept alpha, (RO7239361, anti-myostatin adnectin, RG6206, BMS-986089)	Myostatin inhibition	-	Phase I trial completed in 2017 in healthy volunteers with good tolerance	Phase I/II trial (NCT02515669, THUNDERJET) ongoing in the USA. Completion expected in May 2020. Phase II/III trial (NCT03039686, SPITFIRE) ongoing worldwide. Completion expected in December 2024. **Patients also received GCs**.
Follistatin	Myostatin inhibition	Increase in muscle mass, myofiber size, grip strength and tetanic force, decrease in necrosis, fibrosis, and inflammation [100,101,102]	Phase I/II trial (NCT02354781) with rAAV1.CMV.hu- Follistatin344 conducted in the USA was completed in 2017 with good tolerance and some side-effects. **Patients also received GCs**.	-
Pamrevlumab (FG-3019)	CTGF inhibition	Increase in muscle strength and endurance, decrease in apoptosis and fibrosis in *mdx* muscle [105]	-	Phase II (NCT02606136) ongoing in the USA. Completion expected in April 2021. **Patients also received GCs**.

**Table 6:** Overview of glucose metabolism stabilizing molecules: results in *mdx* myotubes (in vitro) or mice (in vivo), finished clinical trials and results, ongoing clinical trials and due dates, and putative molecules.

Molecule	Involved Pathway	Results in *mdx* Myotubes or Mice	Finished Clinical Trials + Results	Ongoing Clinical Trials + Due Date
Metformin (Glucophage^®^, Fortamet^®^, Riomet^®^)	Insulin sensitizer	Reduction of TGF-β1 and fibrosis and increase in twitch and tetanic tension in *mdx* diaphragm. Protection in myotubes from cardiotoxin degeneration [116,117,118].	Phase I clinical trial (NCT02516085) and Phase III (NCT01995032) trial completed, respectively, in 2012 and 2016 showing a slowing of the disease process but no change in motor function [119,120].	-
AdipoRon	Insulin sensitizer	Delay in disease progression, reduced inflammation, increase of the anti-inflammatory cytokine IL-10, protection from T-lymphocyte and M1 macrophage infiltration, switch to the M2 macrophage type, and boost of the myogenic program; idem for DMD human myotubes [123,124,125,126]	-	-
Pharmacological co-inducer of Hsp72: GBP-15	Insulin sensitizer	Increased muscle strength, architecture, and contractile function in the *mdx* diaphragm when given at early age [109,127]	-	-
Idebenone (Catena^®^, Raxone^®^, Sovrima^®^)	Insulin sensitizer	Improved running performance in *mdx* mice [129]	Two Phase II clinical trials (NCT00654784 and NCT00758225, Delphi extension) were completed in 2007 and 2011 in Belgium; a worldwide Phase III clinical trial (NCT01027884) was finished in 2014. These trials showed good results for respiration among DMD patients [130,131,132].	Two phase III clinical trials are ongoing: NCT02814019, SIDEROS, expected for August 2021, and NCT03603288, SIDEROS-E, expected for January 2024.

**Table 7:** Overview of histone deacetylase (HDAC) stabilizing molecules: results in *mdx* myotubes (in vitro) or mice (in vivo), finished clinical trials and results, ongoing clinical trials and due dates, and putative molecules.

Molecule	Involved Pathway	Results in *mdx* Myotubes or Mice	Finished Clinical Trials + Results	Ongoing Clinical Trials + Due Date
Givinostat (S2170, orphan drug)	HDAC inhibitor	Increase in the cross-sectional myofiber area, membrane stability, and endurance; decrease in fibrosis and inflammation in *mdx* muscle [138,139,140].	Phase I/II trial (NCT01761292); completed in 2017 in Italy with good tolerance and a decrease in necrosis, fibrosis, and fatty tissue. **Patients also received GCs** [141].	Phase II/III (NCT03373968) and III (NCT02851797); trials ongoing in Europe and USA. Completion expected in December 2023 and March 2022. **Patients also received GCs**.

**Table 8:** Overview of nuclear receptor stabilizing molecules: results in *mdx* myotubes (in vitro) or mice (in vivo), finished clinical trials and results, ongoing clinical trials and due dates, and putative molecules.

Molecule	Involved Pathway	Results in *mdx* Myotubes or Mice	Finished Clinical Trials + Results	Ongoing Clinical Trials + Due Date
GW501516	PPARbeta/delta agonist	Stimulates utrophin A expression and the sarcolemmal integrity in *mdx* mice [144,145]	-	-
Vamorolone (VBP-15)	MR antagonist	Increase in membrane stability, grip strength, and force; decrease in inflammation in *mdx* muscle [11]	Phase II trials (NCT02760264 and NCT02760277) were conducted worldwide and completed in 2018, showing good tolerance and improved muscle function in DMD boys. **No other GC intake allowed** [146].	Phase II trials (NCT03038399 and NCT03439670) ongoing worldwide and should be completed in April and May 2020, respectively. **No other GC intake allowed**.

## Abbreviations

ACVR2A, ACVR2B	activin receptors IIA and IIB
ALA	alpha lipoic acid
AP-1	activator protein 1
BMP	bone morphogenic proteins
Cx43	connexin 43
CTGF	connective tissue growth factor
CYP3A4	cytochrome P450 3A4
DAMP	damage-associated molecular pattern
Daxx	death-associated protein
DMD	Duchenne muscular dystrophy
FKBP51 and -52	immunophilin FK506-binding protein 51and 52
Fstl1	follistatin-like 1
FOXO	forkhead box O
GCs	glucocorticoids
GLUT4	glucose transporter 4
GR	glucocorticoid receptor
GRE	glucocorticoid receptor elements
HDAC2, 3, 4 and -6	Histone deacetylase 2, 3, 4 and -6
Hop	hsp70-hsp90 organizing protein
Hsp40, 70, 90	heat shock protein 40, 70, 90
IGF-1	insulin-like growth factor-1
IKB	inhibitor of NF-κB
IR	insulin resistance
IRFs	interferon regulatory factors
IRS1	insulin receptor substrate 1
JAK/STAT	Janus kinase/signal transducer and activator of transcription proteins
JIP1	JNK1 inhibitory protein
JNK	c-Jun N-terminal kinase
L-car	L-carnitine
MAPK	p38 mitogen-activated protein
kinasesMR	Mineralocorticoid receptor
miRNA	microRNA
MyoD	myogenin D
MR	mineralocorticoid receptor
NAC	N-acetylcysteine
NBD	NK-κB Essential MOdulator (NEMO)-Binding Domain
NFATc1	nuclear translocation of nuclear factor of activated T cells c1
NF-KB	nuclear factor kappa-light-chain-enhancer of activated B cells
nNOS	neuronal nitric oxide synthase
NR3C1	Nuclear Receptor Subfamily 3 Group C Member 1
P	phosphorylation
Pax3 and -7	paired box 3 and 7
PDTC	pyrrolidine dithiocarbamate
PGC1α	peroxisome proliferator-activated receptor γ coactivator 1-alpha
PI3K/Akt/mTOR	Phosphoinositide 3-kinases/protein kinase B/mammalian target of rapamycin pathway
PKB/Akt	protein kinase B
PPARα and –γ	peroxisome proliferator-activated receptor α and γ
PTB	polypirimidine tract-binding protein
RA	rheumatoid arthritis
RAF/RAS/ERK	Rapidly Accelerated Fibrosarcoma/Ras-extracellular signal-regulated kinase
ROS	reactive oxygen species
S6K1	ribosomal protein S6 kinase beta-1
SMAD3	Mothers Against Decapentaplegic homolog 3
SMAD6	Mothers Against Decapentaplegic homolog 6
SOCS3	suppressor of cytokine signaling 3
TGF-β	transforming growth factor-β
TIMP3	tissue inhibitor of metalloproteinases 3
TLR	Toll-like receptors
VPA	valproic acid
